# Comparison of Clinical Efficacy of Intravenous Acetaminophen with Intravenous Morphine in Acute Renal Colic: A Randomized, Double-Blind, Controlled Trial

**DOI:** 10.1155/2014/571326

**Published:** 2014-08-13

**Authors:** Kambiz Masoumi, Arash Forouzan, Ali Asgari Darian, Maryam Feli, Hassan Barzegari, Ali Khavanin

**Affiliations:** Department of Emergency Medicine, Imam Khomeini Hospital, Ahvaz Jundishapur University of Medical Sciences, Ahvaz 6193673166, Iran

## Abstract

The aim of this study was to compare the clinical efficacy of intravenous acetaminophen with intravenous morphine in acute renal colic pain management. In this double-blind controlled trial, patients aged 18–55 years, diagnosed with acute renal colic, who met the inclusion and exclusion criteria, were randomized into two groups. First, using the visual analogue scale (VAS), intensity of pain was assessed in both groups. Then, one gram of intravenous acetaminophen or 0.1 mg/kg morphine was infused in 100 mL normal saline to either acetaminophen or morphine group. Intensity of pain was reassessed in 15, 30, 45, and 60 minutes according to VAS criteria. Finally, data from 108 patients were analyzed, 54 patients in each group. No significant difference was observed between the two groups in regard to sex (*P* = 0.13), mean age (*P* = 0.54), and baseline visual analogue score (*P* = 0.21). A repeated measure analysis of variance revealed that the difference between the two treatments was significant (*P* = 0.0001). The VAS reduction at primary endpoint (30 min after drug administration) was significantly higher in the acetaminophen group than in the morphine group (*P* = 0.0001). This study demonstrated that intravenous acetaminophen could be more effective than intravenous morphine in acute renal colic patients' pain relief.

## 1. Introduction

Pain is the most common chief complaint in emergency departments [1]. The essential duty of all care providers is to relieve or prevent patients' suffering pain [2]. Renal colic as a result of urolithiasis is a common cause of severe acute pain. Incidence of kidney stone has been increasing in recent years [3]. Renal colic is an acute syndrome of unilateral flank pain, arising from obstruction of urinary tracts. The first action, after ruling out other diagnoses and attention to potential complications, is finding a suitable pain relief technique. Most common drugs used for renal colic pain relief are parenteral opioids and nonsteroidal anti-inflammatory drugs (NSAIDs) [4, 5].

Several clinical trials compared the efficacy of opioids and NSAIDs in the case of pain relief and adverse effects. Majority of these trials found NSAIDs more effective than opioids or at least as effective as them. Although NSAIDs cause less vomiting, nausea, respiratory suppression, and drowsiness in comparison with opioids, they have potential adverse effects such as platelet dysfunction, nephropathy, and increasing cardiovascular mortality in ischemic heart disease [6–8].

Since NSAIDs and opioids have several side effects, they are unavailable in some emergency departments, seeking new drugs with fewer side effects, and an adequate analgesic property is crucial. One of these alternative drugs is intravenous acetaminophen.

Acetaminophen is acetyl-para-aminophenol. It has analgesic and antipyretic properties comparable to those of aspirin or other NSAIDs [9, 10]. Intravenous acetaminophen is approved for pain management and it is recommended to be infused over 15 minutes [11]. There are a number of trials which compare the intravenous acetaminophen to other injectable pain relief agents [12]. To the best of our knowledge, there are only two clinical trials comparing the intravenous acetaminophen to the morphine in acute renal colic management. These trials showed that acetaminophen and morphine have no significant differences in renal colic pain relief 30 minutes after drug infusion [13, 14].

The aim of this study was to investigate whether or not acetaminophen could reduce pain score equally to morphine and to observe pain score in longer duration (60 minute) after drug administration.

## 2. Material and Methods

This single-center prospective randomized double-blind clinical trial was performed in emergency department of our hospital with an annual census of approximately 100000 visits per year, October 2012–January 2013. Patients, aged 18–55 years, diagnosed with acute renal colic based on their chief complaint, history, and physical examination, and, or past medical history of renal stone, were enrolled in the study. In all participants, kidney or urinary tract stones were confirmed by ultrasound or CT scan.

Exclusion criteria were allergy to morphine or acetaminophen, hemodynamic instability, fever greater than 38°C, evidence of peritoneal inflammation, pregnancy or suspected pregnancy, proven or suspected aortic aneurysm or dissection, use of any analgesic drug up to 6 hours before evaluation, heart failure, renal failure, respiratory failure, liver failure, kidney transplant patients, and opioid addiction.

Eligible patients were randomized to receive acetaminophen or morphine in a 1 : 1 ratio using a computer-generated code. The identities of the study drugs were recorded in a document, folded four times, and then covered for allocation concealment. When a patient was enrolled in the trial, a study nurse retrieved one of the drugs from a box. The medication was prepared by the study nurse and administered by the second nurse who was blinded to the purpose of the intervention. The study drugs were identical in color and appearance; therefore, the patients and study physicians were blinded to identity.

First, using 10 centimeter visual analog scale (VAS), all participants were assessed for severity of pain (10 = the worst possible level of pain and 1 = painless). Then, in the acetaminophen group, patients received intravenous acetaminophen (England, commissioned by Cobel Darou-Iran, in 1 gram vials) with a dose of 1 gram in 100 mL normal saline. In the morphine group, 0.1 mg/kg morphine in 100 mL normal saline was infused. Both drugs were infused during 5–10 minutes.

All participants in both groups were assessed for severity of pain according to VAS standards 15, 30, 45, and 60 minutes after drug administration, and differences in pain level of 2 or more VAS units were considered significant. After 30 minutes, if severity of pain was equal to or more than 5 VAS units, 1 *μ*gr/kg intravenous fentanyl was administered to the patient as rescue therapy. After 60 minutes, patients with pain severity of two units or less were discharged from emergency department. If any degree of pain persisted after min 60, a second 1 *μ*gr/kg dose of fentanyl was administered.

This study was approved by the ethics committee of our university and was registered in the Iranian Registration of Clinical Trials. Besides, Institutional Review Board approval was obtained before starting the trial. A written consent was obtained from all participants. The study was in accordance with Helsinki Declaration 1975.

### 2.1. Statistical Analysis

To consider *α* = 0.05, *β* = 0.2, power = 80%, and the final differences between the two groups at least 2 scores on VAS, the sample size was calculated to be at least 20 in each group. Continuous variables were summarized as mean ± SD and categorical variables as ratios. Two-tailed independent *t*-test was carried out to compare quantitative variables with normal distribution and Chi-squared was done for comparing qualitative ones. A repeated measure analysis was used to compare the differences between the two treatments across the time.

## 3. Results

**Figure 1:**
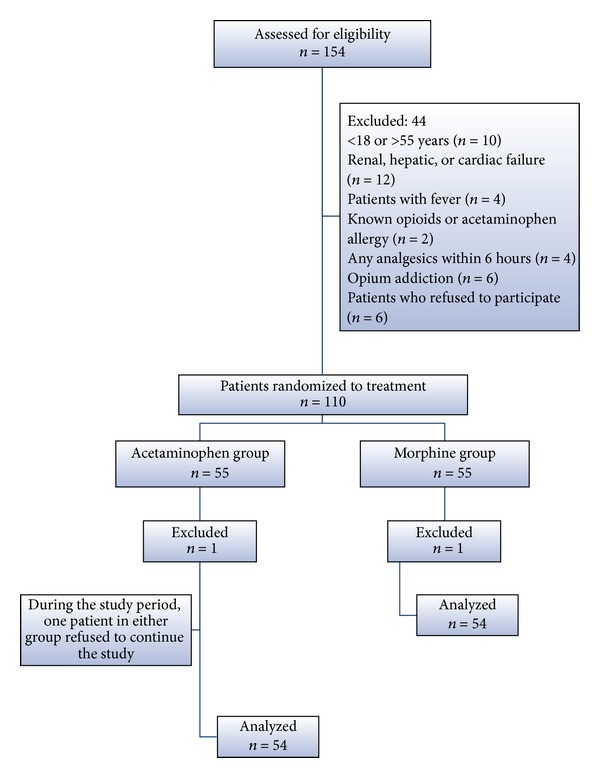
Patient flowchart.

Initially, 154 potential study candidates were enrolled; however, 44 subjects did not meet the inclusion and exclusion criteria. Eventually, 110 patients were allocated randomly between the two groups and data from these participants were analyzed (Figure 1).

There were no significant differences between subjects of the two groups with regard to age, sex, and baseline VAS (Table 1).

**Figure 2:**
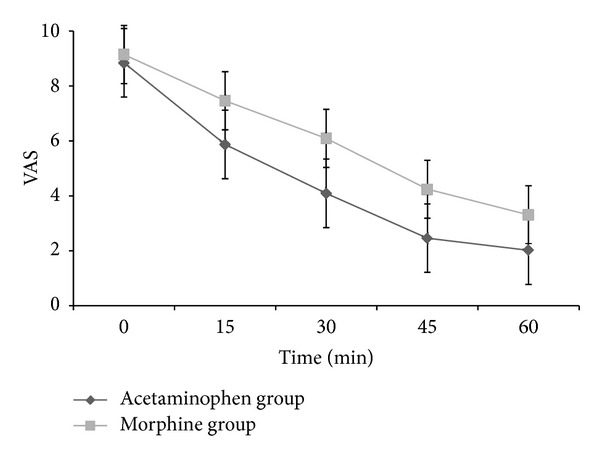
Comparison of visual analogue scores (VAS) between the acetaminophen and the morphine group before and 15, 30, 45, and 60 minutes after drug administration.

The mean ± SD scores of two groups of patients are shown in Table 2. A repeated measure analysis of variance revealed that the difference between the two treatments was significant (*P* = 0.0001) (Figure 2).

The VAS reduction at endpoint (30 min after drug administration) was significantly higher in the acetaminophen group than in the morphine group (4.65 ± 2.25 versus 2.95 ± 2.18, *P* = 0.0001). There was a significant difference between two groups in terms of rescue treatment by fentanyl in min 30 (VAS ≥ 5), 31% versus 55% in the acetaminophen group and the morphine group, respectively (*P* = 0.01). Besides, higher percentages of patients were discharged in min 60 (VAS ≤ 2) in the acetaminophen group (90.7%) than in the morphine group (72.2%) (*P* = 024). Moreover, there were no adverse effects in the acetaminophen group apart from restlessness in 3 patients which was eliminated by slowing infusion rate, while 8 patients in the morphine group had nausea and 6 had vomiting.

## 4. Discussion

Acetaminophen and NSAIDs have some similar pharmacological activities, although acetaminophen is not considered as an NSAID because it does not exhibit significant anti-inflammatory activity. It is, on average, a weaker analgesic than NSAIDs or COX-2 selective inhibitors [15]. To date, the mechanism of action of acetaminophen is not completely understood [10]. The mechanism of action is complex and includes the effects of both the peripheral (COX inhibition) and central (COX, serotonergic descending neuronal pathway, L-arginine/NO pathway, cannabinoid system) antinociception processes and “redox” mechanism [16].

It is now generally accepted that it inhibits COX-1 and COX-2 through metabolism by the peroxidase function of these isoenzymes. Paracetamol often appears to have COX-2 selectivity. The apparent COX-2 selectivity of action of paracetamol is shown by its poor antiplatelet activity and good gastrointestinal tolerance in addition to other studies. It shows selectivity for inhibition of the synthesis of PGs and related factors when low levels of arachidonic acid and peroxides are available, but conversely, it has little activity at substantial levels of arachidonic acid and peroxides. The result is that paracetamol does not suppress the severe inflammation but does inhibit the lesser inflammation and is also active in a variety of inflammatory tests in experimental animals [10, 15, 17, 18]. Acetaminophen reduces the oxidized form of the COX enzyme, preventing it from forming proinflammatory chemicals [19]. This leads to a reduced amount of prostaglandin E2 in the CNS, thus lowering the hypothalamic set point in the thermoregulatory centre [20]. Metabolites of acetaminophen have been found in the spinal cord to suppress the signal transduction from the superficial layers of the dorsal horn to alleviate pain. Acetaminophen also modulates the endogenous cannabinoid system. Its metabolite inhibits the reuptake of the endogenous cannabinoid/vanilloid anandamide by neurons. Anandamide reuptake would result in lower synaptic levels and less activation of the main pain receptor [21].

The half-life of its intravenous form is 2.4 hours and reaches a peak of 15 minutes after infusion [12]. Previously, this drug was used in oral or rectal form, and now its parenteral form is also available in the market. Parenteral form of the drug is available in Iran in 1 gram vials (England, commissioned by Cobel Darou Company-Iran).

Intravenous acetaminophen has been used for postoperative pain management and different types of pain relief in the emergency department [22, 23]. In a comprehensive investigation by Jones in 2011, effectiveness of intravenous acetaminophen was at least equal to that of intravenous morphine in acute renal colic, oral ibuprofen after cesarean section, and oral acetaminophen after CABG and comparable to intramuscular pethidine following tonsillectomy in children [12]. Another study in 2011 by Grissa et al. compared the efficiency of intravenous acetaminophen with intramuscular piroxicam in a not-blinded fashion. Their results showed that intravenous acetaminophen is more effective in renal colic pain relief than that of the piroxicam [24].

In 2009, in a randomized, double-blind, placebo-controlled clinical trial by Bektas et al., the efficacy of acetaminophen, morphine, and placebo was compared in 146 patients with acute renal colic, and it was concluded that intravenous paracetamol is an effective and safe drug for pain control in acute renal colic patients in emergency departments and is comparable with morphine. The mean reduction of VAS pain intensity score was 43 mm in the paracetamol and 40 mm in the morphine group (*P* = 0.74). Besides, in the case of adverse events, two groups showed no significant difference (*P* = 0.14). The study also showed that intravenous paracetamol had a more pronounced effect than morphine at min 15 [13]. Serinken et al. in 2012 showed that both intravenous paracetamol and morphine were similarly effective in pain reduction of renal colic patients at 15 min and 30 min after drug administration. In morphine group, there were more adverse events than in the paracetamol group (14.3% versus 5.3%) [14]. None of the above studies observed patients after minute 30 of drug administration.

The results of our study showed that intravenous acetaminophen could reduce VAS pain score more than morphine; this effect was started at 15 min after drug administration and is constituted during 60 minutes of observation. Although previous studies are not in a line with ours in case of drug efficacy, less adverse effects of acetaminophen are confirmed in both previous and present studies.

### 4.1. Limitations

In the present study, we were supposed to administrate additional pain rescue (fentanyl) in patients with VAS ≥ 5 at min 30. Hence, although pain scores in min 45 and 60 were recorded, the results were confounded by the effect of fentanyl. This could be considered as our study limitation. Another limitation of our study was administrating the acetaminophen during 5–10 minutes, while the recommended method is infusing in 15 minutes. This could be considered as the potential cause of restlessness in acetaminophen group patients.

## 5. Conclusions

Given the above results, in cases of acute renal colic, intravenous acetaminophen could be considered to be a suitable alternative to other available drugs or as a complement to them, in order to reduce their dosage, leading to less potential side effects, and lower costs. The use of intravenous acetaminophen in management of pain induced by other diseases should also be investigated, in order to extend its uses.

## Figures and Tables

**Table 1:** Baseline characteristics of acetaminophen and morphine group participants.

	Acetaminophen group (*n* = 54)	Morphine group (*n* = 54)	*P* value
Sex (male)∗	43 (79.6)	39 (72.2)	0.13
Age (y)∗∗	36.07 (9.7)	34.96 (8.94)	0.54
Baseline∗ VAS	8.84 (1.37)	9.14 (1.13)	0.21

∗Numbers (%) and ∗∗mean (standard deviation).

**Table 2:** Mean ± SD of VAS in two groups across the time.

	Acetaminophen group (*n* = 54)	Morphine group (*n* = 54)	*P* value
Min 15	5.87 ± 2	7.46 ± 2.51	0.0001
Min 30	4.09 ± 2.68	6.09 ± 2.69	0.0001
Min 45	2.46 ± 2.09	4.26 ± 2.51	0.0001
Min 60	2.02 ± 2.03	3.31 ± 2.51	0.004

## References

[1] Ducharme J (2000). Acute pain and pain control: state of the art. *Annals of Emergency Medicine*.

[2] Fosnocht DE, Swanson ER, Bossart P (2001). Patient expectations for pain medication delivery. *The American Journal of Emergency Medicine*.

[3] Marx JA, Hockberger RS, Walls RM, Adams JG (2010). *Rosen's Emergency Medicine: Concepts and Clinical Practice*.

[4] Hetherington J, Philp N (1986). Diclofenac sodium versus pethidine in acute renal colic. *The British Medical Journal*.

[5] Holdgate A, Pollock T (2004). Systematic review of the relative efficacy of non-steroidal anti-inflammatory drugs and opioids in the treatment of acute renal colic. *British Medical Journal*.

[6] Labrecque M, Dostaler L-P, Rouselle R, Nguyen T, Poirier S (1994). Efficacy of nonsteroidal anti-inflammatory drugs in the treatment of acute renal colic. A meta-analysis. *Archives of Internal Medicine*.

[7] Phillips E, Hinck B, Pedro R (2009). Celecoxib in the management of acute renal colic: a randomized controlled clinical trial. *Urology*.

[8] McCormack K, Brune K (1991). Dissociation between the antinociceptive and anti-inflammatory effects of the nonsteroidal anti-inflammatory drugs. A survey of their analgesic efficacy. *Drugs*.

[9] Bertolini A, Ferrari A, Ottani A, Guerzoni S, Tacchi R, Leone S (2006). Paracetamol: new vistas of an old drug. *CNS Drug Reviews*.

[10] Hinz B, Cheremina O, Brune K (2008). Acetaminophen (paracetamol) is a selective cyclooxygenase-2 inhibitor in man. *The FASEB Journal*.

[11] Needleman SM (2013). Safety of rapid intravenous of infusion acetaminophen. *Proceedings (Baylor University Medical Center)*.

[12] Jones VM (2011). Acetaminophen injection: a review of clinical information. *Journal of Pain and Palliative Care Pharmacotherapy*.

[13] Bektas F, Eken C, Karadeniz O, Goksu E, Cubuk M, Cete Y (2009). Intravenous paracetamol or morphine for the treatment of renal colic: a randomized, placebo-controlled trial. *Annals of Emergency Medicine*.

[14] Serinken M, Eken C, Turkcuer I, Elicabuk H, Uyanik E, Schultz CH (2012). Intravenous paracetamol versus morphine for renal colic in the emergency department: a randomised double-blind controlled trial. *Emergency Medicine Journal*.

[15] Graham GG, Davies MJ, Day RO, Mohamudally A, Scott KF (2013). The modern pharmacology of paracetamol: therapeutic actions, mechanism of action, metabolism, toxicity and recent pharmacological findings. *Inflammopharmacology*.

[16] Jóźwiak-Bebenista M, Nowak JZ (2014). Paracetamol: mechanism of action, applications and safety concern. *Acta Poloniae Pharmaceutica*.

[17] Viswanathan AN, Feskanich D, Schernhammer ES, Hankinson SE (2008). Aspirin, NSAID, and acetaminophen use and the risk of endometrial cancer. *Cancer Research*.

[18] Altinoz MA, Korkmaz R (2004). NF-*κβ*, macrophage migration inhibitory factor and cyclooxygenase-inhibitions as likely mechanisms behind the acetaminophen- and NSAID-prevention of the ovarian cancer. *Neoplasma*.

[19] Aronoff DM, Oates JA, Boutaud O (2006). New insights into the mechanism of action of acetaminophen: its clinical pharmacologic characteristics reflect its inhibition of the two prostaglandin H_2_ synthases. *Clinical Pharmacology & Therapeutics*.

[20] Andersson DA, Gentry C, Alenmyr L (2011). TRPA1 mediates spinal antinociception induced by acetaminophen and the cannabinoid Δ^9^-tetrahydrocannabiorcol. *Nature Communications*.

[21] Högestätt ED, Jönsson BAG, Ermund A (2005). Conversion of acetaminophen to the bioactive *N*-acylphenolamine AM404 via fatty acid amide hydrolase-dependent arachidonic acid conjugation in the nervous system. *The Journal of Biological Chemistry*.

[22] Sinatra RS, Jahr JS, Reynolds LW, Viscusi ER, Groudine SB, Payen-Champenois C (2005). Efficacy and safety of single and repeated administration of 1 gram intravenous acetaminophen injection (paracetamol) for pain management after major orthopedic surgery. *Anesthesiology*.

[23] Cattabriga I, Pacini D, Lamazza G (2007). Intravenous paracetamol as adjunctive treatment for postoperative pain after cardiac surgery: a double blind randomized controlled trial. *European Journal of Cardio-Thoracic Surgery*.

[24] Grissa MH, Claessens YE, Bouida W (2011). Paracetamol vs piroxicam to relieve pain in renal colic. Results of a randomized controlled trial. *The American Journal of Emergency Medicine*.

